# Case-control study of the epidemiological and clinical features of human adenovirus 55 and human adenovirus 7 infection in children with acute lower respiratory tract infections in Beijing, China, 2008–2013

**DOI:** 10.1186/s12879-018-3520-z

**Published:** 2018-12-07

**Authors:** Lili Xu, Jun Liu, Chunyan Liu, Yali Duan, Yun Zhu, Baoping Xu, Zhengde Xie

**Affiliations:** 1 Beijing Key Laboratory of Pediatric Respiratory Infection Diseases, Key Laboratory of Major Diseases in Children, Ministry of Education, National Clinical Research Center for Respiratory Diseases, National Key Discipline of Pediatrics (Capital Medical University), Beijing Pediatric Research Institute, Beijing Children’s Hospital, Capital Medical University, National Center for Children’s Health, Beijing, China; 2 National Clinical Research Center for Respiratory Diseases, Resiratory Department, Beijing Children’s Hospital, Capital Medical University, National Center for Children’s Health, Beijing, China

**Keywords:** HAdV-55, HAdV-7, Case-control study, Epidemiological feature, Clinical feature

## Abstract

**Background:**

In adults, the emerging human adenovirus (HAdV) type 55 (HAdV-55) has been reported to cause more severe cases of adenovirus induced acute lower respiratory tract infections (ALRTIs) compared to other HAdV serotypes (HAdV-3, HAdV-7, HAdV-14). However, there is a dearth of comparative studies in children that address differences in the clinical epidemiological features between HAdV-55 and other HAdV serotypes that can also induce severe infection (such as HAdV-7).

**Methods:**

We conducted a retrospective review of pediatric patients hospitalized at Beijing Children’s Hospital with ALRTI from April 2008 to December 2013 who had adenovirus detected from nasopharyngeal or throat samples by PCR. We further compared pediatric patients infected with HAdV-55 to those infected with HAdV-7 using a case-control methodology by matching each subject with HAdV-55 infection to 4 patients with HAdV-7 infection within 2 months of each HAdV-55 infection. Demographic, clinical, and etiological data were collected and analyzed.

**Results:**

Over the five-year period, HAdV was detected in 194 children. Of these, 8 were HAdV-55 positive. Epidemiological results showed that HAdV-55 infection was observed only in 4% of adenovirus infected children whereas HAdV-7 infection proportioned 53%. Most cases of HAdV-55 infection were identified during March and April, whereas HAdV-7 infection occurred throughout the year. Wheezing was significantly less frequent in the HAdV-55 group. No patients infected with HAdV-55 presented with vomiting or had any underlying disease. Coinfections with other respiratory tract pathogens were frequent among children infected with either HAdV-55 or HAdV-7.

**Conclusions:**

HAdV-55 circulated in Beijing during spring and appeared to cause pediatric respiratory infections that were as severe as HAdV-7 infections. Broader surveillance studies are needed.

## Background

Human adenovirus (HAdV) type 55 (HAdV-55)is an intertypic recombinant virus described originally as genome type 11a and identified from an outbreak of acute respiratory tract infection (ALRTI) in Shanxi Province, China, in 2006 [1]. In 2011, this pathogen apparently re-emerged and caused several cases of severe community-acquired pneumonia in adults in Beijing, China [2]. Research demonstrated that HAdV-55 showed the pathogenic properties of HAdV-14 but exhibited a neutralizing antigen epitope of HAdV-11 [3]. Further whole-genome sequencing verified that HAdV-55 had a HAdV-14 backbone and partial HAdV-11 hexon gene [4]; therefore, this pathogen was renamed HAdV-55 [5]. Comparative studies have shown that the ability of HAdV to cause severe disease may relate to HAdV serotypes. In adults, the emerging HAdV-55 has been reported to cause more severe cases of adenoviral pneumonia compared to other HAdV serotypes (HAdV-3, HAdV-7, HAdV-14) [6, 7]. However, **t**here is a dearth of comparative studies, particularly in children, that address differences in clinical epidemiological features between HAdV-55 and other HAdV serotypes that can also induce severe infection (such as HAdV-7). In this study, we investigated whether HAdV-55 and HAdV-7(the most common adenovirus circulating in China that can cause severe or acute infection) have different epidemiological and clinical profiles in children, to assess if HAdV-55 virus can cause more severe infection than HAdV-7 as observed in adults.

## Methods

### Patient enrollment

We retrospectively enrolled pediatric patients with ALRTIs who were admitted to Beijing Children’s Hospital from April 2008 to December 2013. Patients with signs and symptoms of respiratory tract infection (such as fever, coughing, expectoration), and lower respiratory signs (tachypnea, dyspnea, retractions, or wheezing/rales upon auscultation etc.) were defined as ALRTIs. Chest X-rays were taken for all patients. Nasopharyngeal aspirate or throat swab specimens were collected from each patient in virus transport media and stored at − 80 °C prior to use. No repeated samples were collected from any patient.

For each patient with HAdV-55 infection, we matched four neighbours with HAdV-7 infection as controls. Control subjects were collected within two months before and two months after the HAdV-55 cases. The nearest neighbours were recruited first.

### Clinical information collection

Clinical information was collected using a standardized data form. A clinical scoring system for ALRTIs, which related with hospitalization time, pediatric intensive care unit (PICU) admitted time, oxygen supplemented time, maximal FIO_2_, was used to assess severity of illness [8]. The score values ranged from 0 to 14. The median score value of 7 or greater was used to define severe ALRTIs infection and scores less than 7 were considered as mild or moderate disease.

### Preparation of nucleic acids and detection of pathogens

NucliSens easyMAG system (bioMérieux, Marcy-l’Etoile, France) was used to extract viral nucleic acids in accordance with the manufacturer’s instructions. cDNA was reverse transcribed from RNA using random primers and a SuperScript II reverse transcriptase (Invitrogen). Nineteen common respiratory viral pathogens and subtypes (influenza A (IFA); IFA subtype H1; IFA subtype H3; 2009 H1N1; influenza B (IFB); HAdV; human parainfluenza virus (HPIV) 1–4; respiratory syncytial virus (RSV) A and B; human metapneumovirus (HMPV); enteroviruses and rhinoviruses (EV/RhV); human coronavirus (HCoV) HKU1, 229E, NL63 and OC43; and human bocavirus (HBoV)) were detected by using the Luminex xTAG respiratory viral panel assay and a Luminex 200 instrument (Luminex, Austin, TX). HAdV-positive samples were further amplified using a nested PCR procedure that targeted hypervariable regions 1–6 of the hexon gene, as described previously [9]. PCR products were purified and sequenced for further confirmation. Meanwhile, bronchial alveolar lavage fluids (if available), blood, and cerebrospinal fluid (if available) from HAdV-55 and HAdV-7 infected patients during the entire hospital admission were plated onto selective agar plates to identify any bacteria or fungi.

### Statistical analysis

Data analyses were performed using SPSS 19.0. A two-tailed independent-samples *t*-test method was used to compare continuous variables between two groups. Univariate analysis was conducted using χ^2^ tests or Fisher’s exact test for categorical data. Probability < 0.05 was considered to be statistically significant.

## Results

**Fig. 1:**
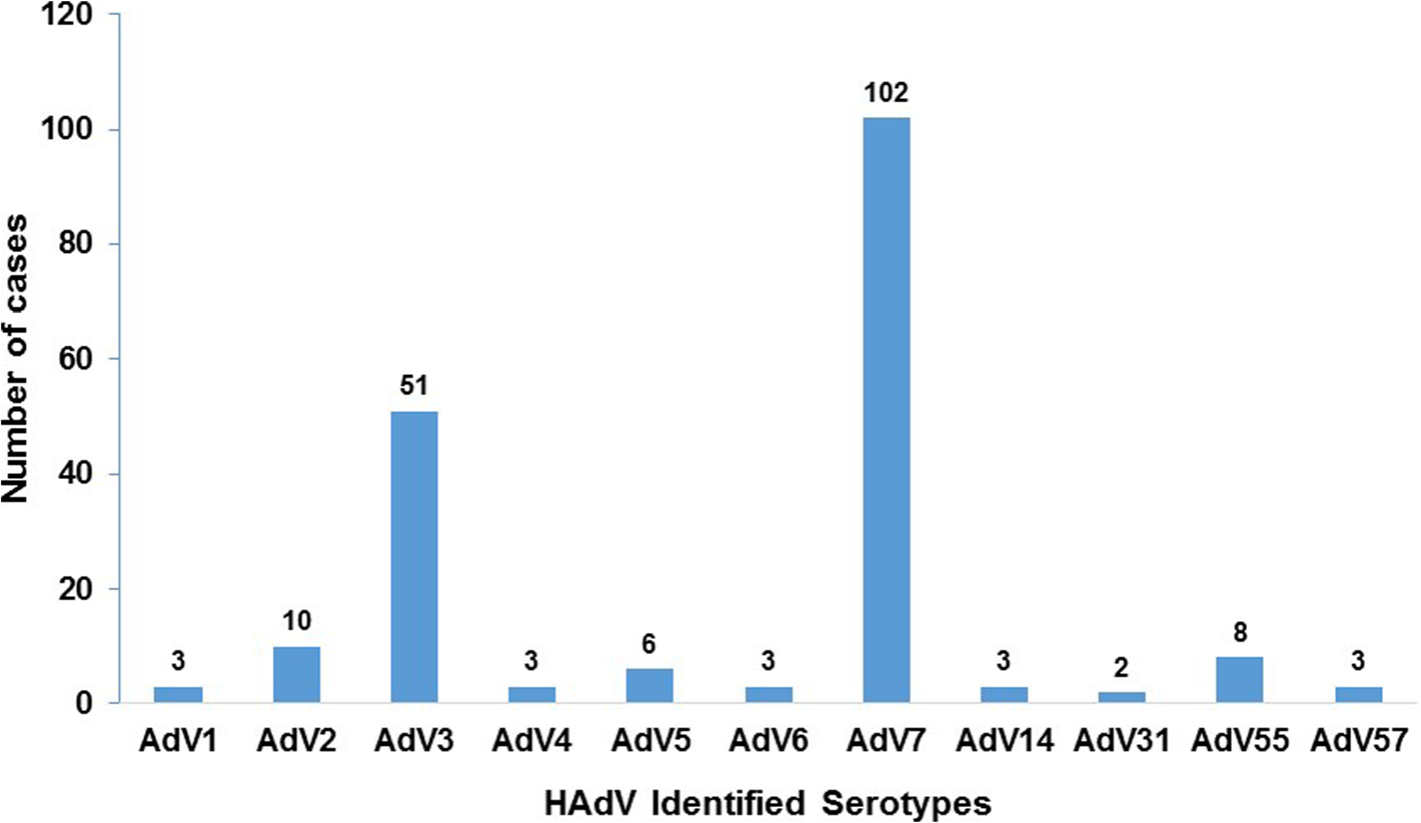
The distribution of HAdV serotypes detected in pediatric ALRTIs cases in Beijing, China, 2008–2013

From April 2008 to December 2013, 3428 pediatric patients with ALRTIs were identified. A total of 194 HAdV-positive cases were detected using the Luminex xTAG respiratory viral panel assay; the most prevalent HAdV serotype was HAdV-7 (102/194, 53%), followed by HAdV-3 (51/194, 26%). Eight cases (8/194, 4%) of HAdV-55 infection were detected. Other newly emerging and re-emergent serotypes or variants, such as HAdV-57 (3/194, 2%) and HAdV-14 (3/194, 2%), were also identified (Fig. 1).

**Fig. 2:**
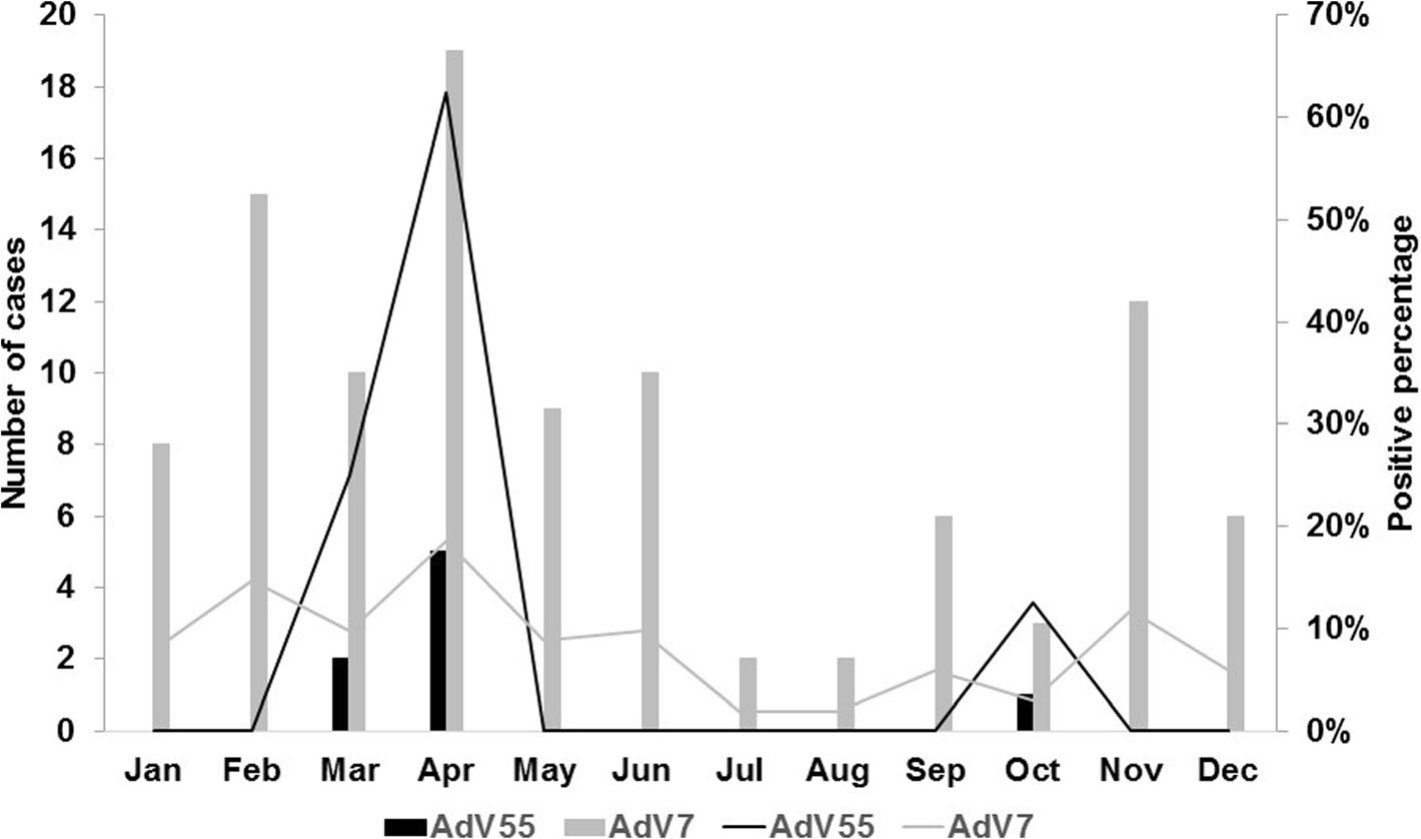
The temporal distribution of HAdV-55 and HAdV-7 detection in pediatric ALRTIs cases in Beijing, China, 2008–2013

Among the 8 HAdV-55 positive cases, 5 (63%) cases were detected in April, 2 (25%) cases were detected in March, and 1 (13%) case was detected in October. In contrast, HAdV-7 infections occurred throughout the year, although such infections were also most frequent in April (19%) (Fig. 2).

Among the 8 HAdV-55 infected patients, one child was an outpatient, and we only knew her age and sex. Another patient left hospital in advance and without recovery, and her clinical features (except for the first blood test after admission to hospital) could not be documented. All 32 matched control subjects were inpatients.

Table 1 shows clinical characteristics of the patients with HAdV-55 infection and the control subjects with HAdV-7 infection. There were no cases of mortality in either group. Most clinical symptoms and signs, and blood parameters did not significantly differ between the two groups, with the exception of wheezing, which was observed in 17% of HAdV-55-infected patients and 63% of HAdV-7-infected patients (*P* = 0.038).

**Table 1:** Clinical features of patients infected with HAdV-55 or HAdV-7 in this study

	HAdV55 (*n* = 8)	HAdV7 (*n* = 32)	*P*
Age (yrs)	0.73 ± 0.78	2.48 ± 3.06	0.120
Male (%)	5/8 (63%)	26/32 (81%)	0.256
Any underlying diseases	0	2/32	0.529
Symptoms and signs			
Fever≥38 °C	7/7 ^*a*^ (100%)	32/32 (100%)	/
Maximum temperature (°C)	39.41 ± 0.94^*b*^	39.79 ± 0.64	0.161
Duration of fever (days)	14.5 ± 10.75^*b*^	15.14 ± 5.48	0.960
Cough	6/6^*b*^ (100%)	32/32 (100%)	/
Rhinorrhea	2/6^*b*^ (33%)	4/32 (13%)	0.199
Wheezing	1/6^*b*^ (17%)	20/32 (63%)	0.038^*c*^
Swelling of tonsils	4/6^*b*^ (67%)	13/32 (41%)	0.239
Rash	2/6^*b*^ (33%)	5/32 (16%)	0.305
Vomiting	0^*b*^	3/32 (9%)	0.435
Diarrhea	1/6^*b*^ (17%)	11/32 (34%)	0.392
Dyspnea	2/6^*b*^ (33%)	10/32 (31%)	0.920
Lung infiltrates	4/6^*b*^ (67%)	16/32 (50%)	0.453
Complication^*d*^	2/7^*a*^ (29%)	18/32 (56%)	0.184
Laboratory detection			
WBC (10^9^/L)	11.48 ± 3.08^*a*^	10.64 ± 5.71	0.709
Neutrophil %	63.20% ± 15.03%^*a*^	57.98% ± 17.46%	0.469
Lymphocyte %	29.26% ± 12.53%^*a*^	35.25% ± 17.59%	0.402
Platelet (10^9^/L)	274.71 ± 184.61^*a*^	291.29 ± 146.87	0.801
CRP (mg/L)	26.53 ± 24.84^*a*^	33.42 ± 45.86	0.706
Treatment			
ICU admission	1/7^*a*^ (14%)	5/32 (16%)	0.929
Mechanical ventilation	1/6^*b*^ (17%)	9/32 (28%)	0.559
Immunoglobulin	2/7^*a*^ (29%)	17/32 (53%)	0.239
Length of hospital stay (days)	11.33 ± 6.86^*b*^	19.10 ± 10.41	0.109
Clinical score^*e*^	6.43 ± 2.44^*a*^	8.21 ± 2.98	0.148

Note:

^a^One subject was an outpatient, and information about this subject was not documented

^b^There was a patient (other than the aforementioned outpatient) who left hospital in advance and without recovery, and her clinical features (except for the first blood test after admission to hospital) could not be documented

^c^Statistically significant (*P* < 0.05)

^d^Complications include respiratory failure, cardiac damage, or liver function damage

^e^A clinical scoring system for ALRTIs was used to assess severity of illness on the day of enrollment

Furthermore, no patient infected with HAdV-55 exhibited vomiting or had any underlying disease; however, 3 (9%) patients vomited during the course of HAdV-7 infection, and 2 HAdV-7-infected patients had underlying disease (congenital heart disease and wheezing). Meanwhile, only 29% (2/7) of HAdV-55 infected patients developed respiratory failure, cardiac damage, or liver function damage, whereas in HAdV-7 infected group, the above mentioned complications arosed in more than half of the patients (56%, 18/32); however, this was not statistically significant (*P* = 0.184).

**Fig. 3:**
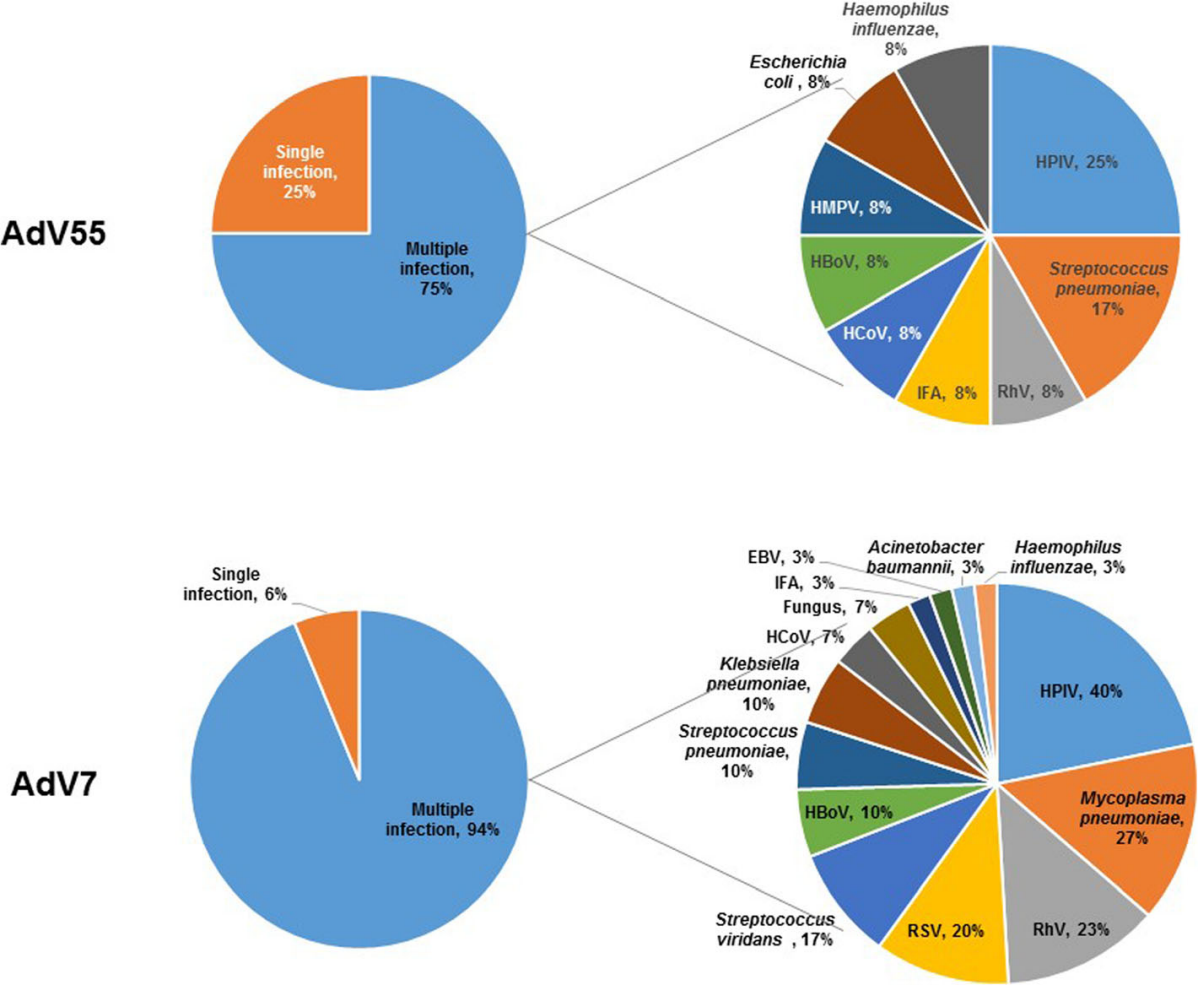
Single and multiple infections involving HAdV-55 or HAdV-7 in pediatric ALRTIs cases in Beijing, China, 2008–2013

Co-infections were observed in 75 and 94% of cases of HAdV-55 and HAdV-7 infections, respectively. HAdV-55 patients were most frequently coinfected with HPIV (25%); *Streptococcus pneumoniae* (17%), RhV (8%), IFA (8%), HCoV (8%), HBoV (8%), HMPV (8%), *Escherichia coli* (8%), and *Haemophilus influenzae* (8%) were also detected in cases involving multiple infection in the HAdV-55 group, in which the percentages of double, triple, and quadruple infection were each 33%. HAdV-7-infected patients were also most commonly coinfected with HPIV (40%); in the HAdV-7 group, multiple infections involving *Mycoplasma pneumoniae* (27%), RhV (23%), RSV (20%), viridans group *Streptococci* (17%), HBoV (10%), *Streptococcus pneumoniae* (10%), *Klebsiella pneumoniae* (10%), HCoV (7%), fungus (7%), IFA (3%), EBV (3%), *Acinetobacter baumannii* (3%), and *Haemophilus influenzae* (3%) were also detected. In that group, the percentages of double, triple, quadruple, quintuple, and sextuple infections were 56, 13, 13, 9, and 3%, respectively (Fig. 3).

## Discussion

Epidemiological results in this study showed that in children, HAdV-55 infection was observed much less frequently than HAdV-7 infection. Most cases of HAdV-55 infection were identified during March and April, whereas HAdV-7 infection occurred throughout the year. These results were in accordance with previous reports about the typical adenoviruses season months [6, 10, 11].

A comparative study involving adult patients was conducted by Cao et al to discern epidemiological and clinical differences between HAdV-55 and other HAdV serotypes (HAdV-7, HAdV-3, HAdV-14, HAdV-50, and HAdV-C) [6]. Their results showed that most HAdV-55 cases were identified during February and March. They also found that patients with HAdV-55 infection had higher pneumonia severity index scores (*P* = 0.030) than patients infected with another HAdV serotype (HAdV-7, HAdV-3, HAdV-14, HAdV-50, or HAdV-C). Sun et al also found that HAdV-55 may cause severe acute respiratory distress syndrome (ARDS) in immunocompetent young men. They conducted a prospective, single-center observational study of pneumonia with ARDS with confirmed HAdV-55 infection in immunocompetent adults, and four (80%) of the five patients died despite receiving appropriate respiratory support [7]. However, our data showed that the HAdV-55 group had lower ALRTI clinical scores than the HAdV-7 group, although this difference was not significant.

Cao and Lu separately reported the presence of underlying diseases in 19.1 and 50% of HAdV-55 patients, respectively [6, 12]. We could not exclude the possibility that vomiting and underlying disease may be observed if our study is enlarged to include additional cases of HAdV-55 infection. One potential weakness of our data is that there were only 8 patients with HAdV-55 infection. However, these patients represented all positive cases of HAdV-55 infection in our hospital from April 2008 to December 2013. This result also indicated that HAdV-55 infection was less common than HAdV-7 infection in children.

Our results were consistent with those reported by Lu, who found that coinfections were frequent and observed coinfection in 83.3% of hospitalized children infected with HAdV-55 [12]. However, our data differed from Cao’s data in certain respects. Their results showed that only 4 (19.1%) HAdV-55-infected patients were coinfected with other pathogens, with *Mycoplasma pneumoniae* detected in three patients and HPIV and IFB detected in one patient, and that only 1 HAdV-7-infected patient exhibited coinfection (with *Mycoplasma pneumoniae*) [6]. These inconsistent findings may be attributable to different pathogen detection methods. The Luminex xTAG respiratory viral panel assay is more sensitive and comprehensive than the approach used by Cao et al. Meanwhile, in our study, a nasopharyngeal aspirate or throat swab specimen was considered to be co-infected with one pathogen when either the Luminex xTAG respiratory viral panel assay or the selective agar plate culture showed positive result. Furthermore, respiratory tract specimens, blood, and cerebrospinal fluid collected during the entire hospital admission from patients were cultured to identify any bacteria or fungi. For these reasons, our study may have identified more co-infections than previous studies.

## Conclusion

To our knowledge, our investigation is the only case-control study to date involving pediatric patients with HAdV-55 or HAdV-7 infection and comparisons of epidemiological and clinical features of HAdV-55-infected subjects and HAdV-7-infected subjects. We concluded that HAdV-55 circulated in Beijing during spring and appeared to cause pediatric respiratory infections that were as severe as HAdV-7 infections. Our data provide new insight into the epidemiology of HAdV-55 infection in pediatric ALRTIs patients. A wider or longer surveillance studies are needed to evaluate the spectrum of disease caused by this emerging pathogen in China.

## Abbreviations

ALRTIs: Acute lower respiratory tract infections
ARDS: Acute respiratory distress syndrome
EV: Enteroviruses
HAdV: Human adenovirus
HBoV: Human bocavirus
HCoV: Human coronavirus
HMPV: Human metapneumovirus
HPIV: Human parainfluenza virus
IFA/IFB: Influenza A/B virus
PICU: Pediatric intensive care unit
RhV: Rhinoviruses
RSV: Respiratory syncytial virus
